# Public faith in science in the United States through the early months of the COVID-19 pandemic

**DOI:** 10.1016/j.puhip.2021.100103

**Published:** 2021-03-16

**Authors:** Daniel Silva Luna, Jesse M. Bering, Jamin B. Halberstadt

**Affiliations:** a Centre for Science Communication, University of Otago, New Zealand; b Faculty of Psychology, University of Otago, New Zealand

**Keywords:** Faith in science, Belief in science, COVID-19, Coronavirus, Trust in science

## Abstract

**Objectives:**

Given the centrality of science over the course of the COVID-19 crisis, we evaluate changes in people’s beliefs in the power of science in the United States over the first four months of the pandemic.

**Study design:**

Post-hoc analysis of cross-sectional survey data.

**Methods:**

A convenience sample of 1327 participants was recruited through Amazon’s Mechanical Turk service for three surveys carried out in 14–25 January, 27 March to 1 April, and 28–29 May of 2020. Respondents completed a ten-item instrument measuring different aspects of their perceptions of science including trust, interest, and faith (answer to the question: “How much do you agree with the following statement: Science can sort out any problem.”). We conducted multivariate analysis of covariance (MANCOVA) with faith, interest, and trust as dependent variables, time as the independent variable, and political orientation and religiosity as between-subjects covariates.

**Results:**

The data revealed that public levels of faith in science increased between January (M ​= ​3.2) and both March (M ​= ​3.42) and May (M ​= ​3.4). By contrast, we observed no changes in interest and trust in science over the same time period.

**Conclusions:**

We speculate that increases in faith in science during the first four months of the pandemic helped people cope with the uncertainty and existential anxiety resulting from this public health crisis.

## Highlights

• We observed public faith in science increase in the United States throughout the first four months of the Covid-19 pandemic.
• Trust and interest in science did not shift over the same time period.
• Increases in faith may have helped people cope with the uncertainty and existential anxiety from this public health crisis.

## 1 Introduction

Economic upheavals, public health emergencies, natural disasters and other instances of public crisis are known to change people’s perceptions of science and science-related issues [1]. Different forms of faith may also change in times of crises. Religious belief, for example, may increase during difficult times, presumably as a coping mechanism to address uncertainties and existential anxieties [2]. Sibley and Bulbulia (2012)[3], for instance, observed increased religious beliefs among earthquake-affected New Zealanders following the natural disaster in Christchurch.

Faith in science, or the “belief in the value of science as an institution and in its superiority as a source of knowledge” [4] may play a similar role to religious faith in attenuating existential concerns and coping with uncertainty. Farias et al. (2013)[4], observed that participants who contemplated their own death showed stronger faith in science than did those in a control group asked to write about their experience with dental pain. Relatedly, people increase their belief in scientific progress when placed in low-control situations [5].

Within the first five months of the COVID-19 pandemic, at least 100,000 people had died from the coronavirus in the United States, over 20 million people lost their jobs, and the worsening effects on mental health, food security, and other aspects of people’s lives revealed the sobering impact of the public health crisis [6]. Besides the unprecedented uncertainty, people also faced rising existential concerns [7]. These factors, which are precisely of the variety underlying increased faith, likely foster a need for control and reassurance.

Considering the unparalleled impact of the novel coronavirus in people’s lives, we sought to examine the effects of this crisis on Americans’ faith in the power of science. The purpose of our original data collection was unrelated to the pandemic. However, the timing of our first two surveys on Americans’ perceptions of science happened to coincide with the period just prior and subsequent to the outbreak of COVID-19 in the United States (January 14–25), and at the height of the initial lockdowns (March 27 – April 1). Realizing the significance of this timing in light of the pandemic, we then administered the survey a third time, at a time point just after the 100,000^th^ coronavirus death in the US was reported (May 28–29). At each of the three points, a medium-sized online sample of participants in the United States completed a ten-item short instrument measuring perceptions of science [8].

We wanted to know if faith in science had changed over the course of a delineable COVID-19 trajectory in the United States. The unique testing period involved three distinct time points for direct comparison, which can be identified roughly as *immediately before* the outbreak, *at the height of* public panic (i.e., as lockdowns were coming increasingly into effect), and *after several months* of Americans living under COVID-19 had passed.

## 2 Methods

A convenience sample of 1327 participants was recruited through Amazon’s Mechanical Turk (MTurk) service for three surveys. We restricted respondents to US-based geolocations and with 95% or more HIT approval rate and excluded those who failed to respond to an attentiveness question (34 participants). Moreover, we excluded data from the fastest 5% of participants, based on their survey completion time, to control for low-quality data such as careless responses (66 participants). After such removals, the final sample consisted of 1227 participants. The three surveys were carried out in 14–25 January (n ​= ​473), 27 March to 1 April (n ​= ​294), and 28–29 May of 2020 (n ​= ​460).^1^ We restricted participants from participating in more than one survey through their MTurk worker ID (see Table 1).

**Table 1:** Sociodemographic characteristics of respondents.

Baseline characteristic	*January (N ​= ​473)* Frequency (%)	*March (N ​= ​294)* Frequency (%)	*May (N ​= ​460)* Frequency (%)	*Total (N ​= ​1227)* Frequency (%)
Gender				
male	258 (54.5)	143 (48.6)	256 (55.7)	657 (53.5)
female	212 (44.8)	149 (50.7)	203 (44.1)	564 (46.0)
other	3 (0.6)	2 (0.7)	1 (0.2)	6 (0.5)
Ethnicity				
Black or African American	73 (15.4)	33 (11.2)	72 (15.7)	178 (12.7)
Hispanic or Latina/o/x	23 (4.9)	17 (5.8)	44 (9.6)	84 (6.0)
White	349 (73.8)	209(71.1)	298 (64.8)	856 (61.1)
Other	28 (6.0)	35 (11.9)	46 (10.0)	109 (20.2)
	*January (N ​= ​473)* *Mean (SD)*	*March (N ​= ​294)* *Mean (SD)*	*May (N ​= ​460)* *Mean (SD)*	*Total (N ​= ​1227)* *Mean (SD)*
Age (years)	38.5 (11.9)	36.8 (10.9)	36.71 (11.2)	37.4 (11.4)
Faith in science - How much do you agree with the following statement? “Science can sort out any problem.” (1 ​= ​strongly disagree, 5 ​= ​strongly agree)	3.20 (1.13)	3.48 (1.14)	3.40 (1.09)	3.34(1.12)
Interest in science - How interested are you in science? (1 ​= ​not at all, 5 ​= ​a great deal)	4.05 (0.96)	4.12 (0.92)	4.07 (0.91)	4.08 (0.93)
Trust in science - How much do you trust science in general? (1 ​= ​not at all, 5 ​= ​a great deal)	4.18 (0.83)	4.21 (0.83)	4.20 (0.77)	4.19 (0.81)

We used an adaptation of the 10-item questionnaire on public perceptions of science developed by Füchslin et al. (2018) [8]. We chose this instrument because it captures a broad range of psychological constructs, including interest, trust, beliefs and reservations about science.

All survey items were presented in a Likert-type end-defined format. Because the original study was unrelated to the COVID-19 crisis, for present purposes we focused our attention on the survey item that best tracked short-term change on the theoretical construct of primary interest: “How much do you agree with the following statement: Science can sort out any problem.” This item is hereafter called *faith in science*. To control for other, related constructs we also analyzed items tapping *interest in science* (“How interested are you in science?”) and *trust in science* (“How much do you trust science in general?”), over the same time period. We also controlled for participants’ political ideology and religious beliefs by including both variables as covariates.

## 3 Results

Our main analysis was conducted using multivariate analysis of covariance (MANCOVA) with faith, interest, and trust as dependent variables, time as the independent variable, and political orientation and religiosity as between-subjects covariates. The results of the multivariate analysis was statistically significant for time with Wilks’ Λ: a ​= ​0.988, (F(6, 2440) ​= ​2.377, *p* ​= ​.027, ηp2 ​= ​0.006). Both religiosity, Wilks’ Λ: a ​= ​0.979, (F(3, 1220) ​= ​8.93, *p* <.001, ηp2 ​= ​0.021) and political orientation, Wilks’ Λ: a ​= ​0.962, (F(3, 1220) ​= ​16.06, *p* ​< ​.001, ηp2 ​= ​0.038) were also statistically significant predictors of the combined dependent variables.

Follow up between-subjects effects tests using analysis of variance (ANOVA) revealed no significant differences between the time periods for interest and trust (F(2,1222) ​= ​0.599, *p* ​= ​.550) and (F(2, 1222) ​= ​1.233, *p* ​= ​.292), respectively. However, group differences for time were observed for faith (F(2, 1222) ​= ​8.326, *p* ​= ​0.001, ηp2 ​= ​0.011). Moreover, post hoc multiple comparisons (Bonferroni corrected) indicate that faith in science was significantly lower in January (M ​= ​3.20) than in March (M ​= ​3.48, *p* ​= ​0.003) or May (M ​= ​3.40, *p* ​= ​.014), while the difference between March and May was not statistically significant (*p* ​= ​1.000).

## 4 Discussion

Faith in science increased significantly over the first two months of the COVID-19 pandemic in the United States and then stayed relatively flat over the subsequent two months. By contrast, other aspects of people’s relationship to science, including interest and trust in science, remained unchanged over the same time period. These latter findings are similar to those reported recently by Agley (2020) [10], who found no differences in people’s trust in science before and after the pandemic began in the United States.

Stenmark (1997) [9] has argued that the belief that “science can sort out any problem” is akin to a comprehensive form of scientism. Such an ideological stance reflects people’s perception of science’s superiority; a type of faith that could complement or replace other forms of comforting beliefs such as those derived from religion [4]. Considering the existential concerns and uncertainties resulting from the many changes to daily life as a result of the COVID-19 crisis, and the potential of a scientific solution to this pandemic (e.g., treatments, vaccines), it is perhaps unsurprising that our post-hoc analyses revealed that people’s faith in science has increased.

Our findings are consistent with controlled studies showing that, when participants are confronted with their own mortality [4], or placed under increasing levels of uncertainty [5] they can turn to science for solace. Similarly, other research has shown that irrespective of one’s secular commitments, people turn increasingly to religious beliefs in times of public crisis [3]. In fact, our data are perhaps the first to show that faith in science increases following a public health crisis. Even after controlling for different political orientations and varying degrees of religiosity, we observed a rise in faith in science.

Considering the convenience sampling used (i.e., MTurk) we should be cautious with the generalizability of the data. Additionally, because we used single Likert-type items for the original purposes of our survey, our conceptualization of constructs such as faith in science may not fully capture their multidimensional nature; this may have been better achieved by the use of a validated scale for such constructs [4]. Moreover, our data collection did not include questions about people’s existential worries or perceptions of uncertainty that could have helped disentangle the precipitating forces driving increases in faith in science. Although this attenuating role of faith in science is a reasonable interpretation, it is admittedly speculative, given that we could only track changing faith in science over the course of this unique historical period.

COVID-19 is arguably the most significant public health crisis that contemporary Americans have experienced. In our dataset, with sampling occurring at key points in the trajectory of the coronavirus’s spread, we observed an overall increase in faith in science in the four months since the start of the current pandemic, with people generally becoming more strongly convinced of the problem-solving powers of science. Overall trust and interest in science, however, remained stable. Although we cannot infer causality from the present study, similar studies report convergent results [10]. Continued tracking of these data points will enable researchers to determine if these changes in people’s perceptions of science are short- or long-lived, mapping them to ongoing developments in the fight against COVID-19.

## Ethical approval

Approved by the Human Ethics Committee of the University of Otago #D19/374, #D20/065, and #D20/152.

## Funding

This research did not receive any specific grant from funding agencies in the public, commercial, or not-for-profit sectors.

## Declaration of competing interest

The authors declare that they have no known competing financial interests or personal relationships that could have appeared to influence the work reported in this paper.

## Footnotes

1 The data were collected as part of a series of studies on the nature of emotions in science communication. In the first and third studies (i.e., January and May 2020), participants performed a word-association task. In the second study (i.e., March), participants were asked to recall an emotional experience and write about it. The difference in sample sizes is the result of budget constraints imposed by task length.
